# Costs of administering injectable contraceptives through health workers and self-injection: evidence from Burkina Faso, Uganda, and Senegal^☆^^☆☆^

**DOI:** 10.1016/j.contraception.2018.05.018

**Published:** 2018-11

**Authors:** Laura Di Giorgio, Mercy Mvundura, Justine Tumusiime, Allen Namagembe, Amadou Ba, Danielle Belemsaga-Yugbare, Chloe Morozoff, Elizabeth Brouwer, Marguerite Ndour, Jennifer Kidwell Drake

**Affiliations:** a PATH, PO Box 900922, Seattle, WA 98109, USA; b PATH, PO Box 7404, Kampala, Uganda; c Independent consultant, Cite Menthor Diouf Villa N. 02 Zac Mbao, Dakar, Senegal; d Institut de Recherche en Sciences et de la Santé, P.O. Box 7192, Ouagadougou, 03, Burkina Faso; e University of Washington, 1959 NE Pacific Street, Seattle, WA 98195, USA; f PATH, BP 15115, Dakar-Fann, Dakar, Senegal

**Keywords:** Costs, DMPA-SC, Contraceptive service delivery costs, Injectable contraception, Self-injection, Community-based distribution

## Abstract

**Objective:**

To evaluate the 12-month total direct costs (medical and nonmedical) of delivering subcutaneous depot medroxyprogesterone acetate (DMPA-SC) under three strategies — facility-based administration, community-based administration and self-injection — compared to the costs of delivering intramuscular DMPA (DMPA-IM) via facility- and community-based administration.

**Study design:**

We conducted four cross-sectional microcosting studies in three countries from December 2015 to January 2017. We estimated direct medical costs (i.e., costs to health systems) using primary data collected from 95 health facilities on the resources used for injectable contraceptive service delivery. For self-injection, we included both costs of the actual research intervention and adjusted programmatic costs reflecting a lower-cost training aid. Direct nonmedical costs (i.e., client travel and time costs) came from client interviews conducted during injectable continuation studies. All costs were estimated for one couple year of protection. One-way sensitivity analyses identified the largest cost drivers.

**Results:**

Total costs were lowest for community-based distribution of DMPA-SC (US$7.69) and DMPA-IM ($7.71) in Uganda. Total costs for self-injection before adjustment of the training aid were $9.73 (Uganda) and $10.28 (Senegal). After adjustment, costs decreased to $7.83 (Uganda) and $8.38 (Senegal) and were lower than the costs of facility-based administration of DMPA-IM ($10.12 Uganda, $9.46 Senegal). Costs were highest for facility-based administration of DMPA-SC ($12.14) and DMPA-IM ($11.60) in Burkina Faso. Across all studies, direct nonmedical costs were lowest for self-injecting women.

**Conclusions:**

Community-based distribution and self-injection may be promising channels for reducing injectable contraception delivery costs. We observed no major differences in costs when administering DMPA-SC and DMPA-IM under the same strategy.

**Implications:**

Designing interventions to bring contraceptive service delivery closer to women may reduce barriers to contraceptive access. Community-based distribution of injectable contraception reduces direct costs of service delivery. Compared to facility-based health worker administration, self-injection brings economic benefits for women and health systems, especially with a lower-cost client training aid.

## 1 Introduction

More than 225 million women in low- and middle-income countries (LMIC) have an unmet need for modern contraceptives, the largest need being among women living in rural areas [1]. New contraceptive technologies and delivery strategies may reduce barriers to family planning access and continuation, thereby addressing unmet need.

Subcutaneous depot medroxyprogesterone acetate (DMPA-SC) is a novel formulation and presentation of the injectable DMPA. The DMPA-SC product available to Family Planning 2020 countries is Pfizer’s Sayana® Press, which delivers the contraceptive drug through the BD Uniject™ injection system, allowing for easier administration by lay health workers with minimal training and for women to self-inject. Recent studies demonstrated the operational feasibility of these administration modalities and acceptability to women and health workers [2], [3], [4]. Previous formative research in Senegal and Uganda found that DMPA-SC might have logistical benefits relative to intramuscular DMPA (DMPA-IM), but actual costs of delivering DMPA-SC have not yet been evaluated [5]. Given that DMPA-SC is a new contraceptive intervention, there is need to assess any associated increase or decrease in the economic cost of service delivery for both health systems and women compared to existing interventions in order to inform decisions about contraceptive method mix.

Evidence on the costs of contraceptive service delivery in LMIC is generally scarce. Only a few studies have estimated the costs of delivering injectable contraceptives — mainly DMPA-IM [6], [7], [8], [9], [10], [11], [12], [13] — in LMIC, and none have evaluated women’s travel and opportunity costs attributable to seeking contraceptive services. Only one study assessed the costs of delivering DMPA-SC: an analysis of facility-based contraceptive delivery costs in Kenya [13]. Research gaps remain regarding community-based distribution and self-injection of DMPA-SC.

Therefore, we sought to investigate whether the costs to administer DMPA-SC differed from the costs to administer DMPA-IM and whether these costs differed by delivery strategy. We conducted these costing studies in parallel with studies evaluating the method continuation rates of DMPA-SC under facility-based administration in Burkina Faso, community-based distribution via Village Health Teams in Uganda, and self-injection in Senegal and Uganda, all compared to DMPA-IM [14], [15] [Jane Cover, personal communication, 2017]. These studies found no major differences in continuation rates between DMPA-SC and DMPA-IM when the delivery strategy was the same [14], though self-injection of DMPA-SC led to higher continuation rates compared to facility-based delivery of DMPA-IM [15].

The main objective of this study was to assess the costs of delivering DMPA-SC and DMPA-IM using different strategies in three sub-Saharan African countries: Burkina Faso, Uganda and Senegal. We reported costs from a health system perspective and also accounted for women’s travel and time costs to travel to, wait for and receive services. Specifically, we sought to understand the total direct costs of delivering DMPA-SC and DMPA-IM, including commodity costs; costs of provider time, medical supplies and drugs for the treatment of side effects; and travel and time costs to women. We did not seek to directly compare results across countries. However, we can draw some lessons by looking at the costs by delivery strategy, especially in Uganda where we assessed three delivery approaches (i.e., facility-based health worker administration, community-based health worker administration and self-injection) in two studies conducted in a very similar setting (same health care system, unit prices, time period, and partially overlapping geographic areas).

We then used the cost estimates as input in a follow-up cost-effectiveness study [16] which included the impact of discontinuation on pregnancy outcomes and costs.

## 2 Methods

We received in-country approval for conducting the costing studies from the Comité d’Ethique pour la Recherche en Santé in Burkina Faso, Mulago Research Ethics Committee of Uganda and Comité National d’Ethique pour la Recherche en Santé of Senegal. We obtained consent to participate in this study from each health worker interviewed.

### 2.1 Injectable contraceptive service delivery in Burkina Faso, Uganda and Senegal

Burkina Faso introduced DMPA-SC through the facility-based delivery strategy in place for DMPA-IM [17]. Uganda introduced DMPA-SC through community-based distribution by Village Health Teams, most of whom were already providing DMPA-IM and other short-term contraceptive methods [17]. The Village Health Teams were affiliated with health facilities for reporting and replenishing the contraceptive commodities. Finally, Uganda and Senegal piloted DMPA-SC for self-injection under a research setting. A study health worker (nurse or midwife) trained women visiting health facilities and interested in self-injection to self-inject, practicing the technique on a prosthetic. The health worker then observed those deemed proficient in the self-injection technique during their first self-injection. Afterward, the health worker gave the client a training aid, a calendar to assist with reinjection dates and three DMPA-SC units to take home to self-inject. In the facilities participating in the self-injection research study, health workers also administered DMPA-IM.

### 2.2 Costing study design

We conducted four microcosting studies across the three countries. Microcosting is a cost estimation method that involves collecting detailed data on the resources used (input quantities) and the value of those resources (input prices) in the delivery of a health service [18], [19], [20]. Microcosting is particularly useful in the estimation of costs of new interventions or interventions that include nonmarket goods (e.g., volunteer labor), or for studying cost variation within the same procedure [21]. We used structured costing questionnaires to interview health workers on resources used to deliver all contraceptive services. We used a cross-sectional design, whereby we visited each health facility once within the data collection period. Health facilities included in the costing studies were a subsample of the study sites selected in parallel continuation studies using purposive sampling. Table 1 shows information on the study sites and health workers interviewed.

**Table 1:** Study sites, DMPA delivery strategy and sample size for the costing studies

	Burkina Faso	Uganda		Senegal
DMPA-IM service delivery strategy	Facility-based delivery by nurses (routine delivery)	Community-based distribution by Village Health Teams (routine delivery)	Facility-based delivery by nurses or midwives (routine delivery)	Facility-based delivery by nurses or midwives (routine delivery)
DMPA-SC service delivery strategy	Facility-based delivery by nurses (routine delivery)	Community-based distribution by Village Health Teams (routine delivery)	Self-injection (research intervention; first injection supervised at the health facility by a nurse or midwife, subsequent injections administered independently outside the facility setting)	Self-injection (research intervention; first injection supervised at the health facility by a nurse or midwife, subsequent injections administered independently outside the facility setting)
Number of health workers interviewed	30 health workers from 30 facilities across 88 districts	45 Village Health Teams affiliated with 15 facilities across 6 pilot districts	10 health workers from 10 facilities across 5 districts	10 health workers from 10 facilities across 8 districts
Data collection period for health worker interviews	February–March 2016	February–March 2016	September–October 2016	January 2017
Number of women interviewed regarding travel and opportunity costs	990	1224	1161	1294
Data collection period for client interviews	December 2015–April 2016	December 2015–April 2016	April–July 2016	September 2016–January 2017

The direct medical costs for service delivery included the costs of contraceptive commodities, health worker time to deliver family planning services (including time for medical consultation if the client visited the health facility for side effects), medical supplies and drugs for the treatment of side effects. For self-injection, we also included the resources used for training women to self-inject: health worker time to train the client and necessary supplies [practice units, prosthetic (i.e., salt-filled condom), client training aid and reinjection calendar].

In addition, we estimated the direct nonmedical costs (women’s travel costs and time) by interviewing women enrolled in the continuation studies (Table 1). To this purpose, we asked women who agreed to participate in the DMPA continuation studies about their modes of transport, travel time to reach the facility and transport costs.

We estimated the economic costs of contraceptive service delivery to account for donated health commodities as well as for the time of volunteers involved in contraceptive service delivery (Village Health Teams). We estimated annual costs per couple years of protection (CYP) — equivalent to receiving four injections of these 3-month injectable contraceptives [22].

### 2.3 Methods for estimating direct medical costs of service delivery and women’s travel and time costs

Tables 2 and 3 show unit prices. Table 4 shows key input data used to estimate the costs of service delivery. We estimated annual commodity costs per CYP (i.e., by multiplying unit costs by four).

**Table 2:** Unit prices for selected commodities and supplies^a^

	Unit of measure	Unit price
Injectable contraceptives		
Sayana Press (DMPA-SC)	Each	$0.85
DMPA-IM and syringe	Each	$0.83
Supplies		
Male condom	each	$0.05
Safety box (5-L)	each	$0.98
Pregnancy test	each	$0.40
Self-injection training supplies		
Calendar	each	$0.06
Booklet	each	$2.00
Instruction sheet	each	$0.07
Water-filled Uniject injection device	each	$0.30
Salt-filled condom	each	$0.03

a Unit prices did not differ by country.

**Table 3:** Unit prices for selected supplies and resources^a^

	Unit of measure	Burkina Faso	Uganda	Senegal
Supplies				
Pregnancy test	each	$0.40	$0.40	$2.42
Examination gloves	pair	$0.05	$0.16	$0.21
Drugs used for treating side effects				
Paracetamol tablet	500 mg	$0.01	$0.01	$0.01
Ibuprofen tablet	400 mg	$0.02	$0.01	$0.01
Oral contraceptives	1 cycle	$0.51	$0.51	$0.42
Salaries and opportunity costs				
Registered nurse or midwife	Annual salary range	$3418−$5512	$1210−$2397	$4845−$5002
Nursing assistant	Annual salary	NA	$782	NA
Client	Annual opportunity costs based on country’s GDP per capita^b^	$663	$696	$1055

Abbreviations: GDP, gross domestic product; NA, not applicable.

a For the prices of drugs and supplies in Senegal and Burkina Faso, we used values from the national price lists, while in Uganda, we used prices from the Joint Medical Store.

b Adjusted by the percentage of women working.

**Table 4:** Input data used to estimate the costs of contraceptive service delivery

	Burkina Faso: facility-based administration of DMPA-SC and DMPA-IM	Uganda: community-based administration of DMPA-SC and DMPA-IM	Uganda: self-injection of DMPA-SC and facility-based administration of DMPA-IM	Senegal: self-injection of DMPA-SC and facility-based administration of DMPA-IM
Health workers self-reported time use				
First visit (min)	29 (15−60)	39 (28−50)	27 (15−45)	39 (10−53)
Follow-up visits for women not experiencing side effects (min)	13 (5−25)	19 (14−23)	19 (14−23)	10 (3−30)
Follow-up visits for women experiencing side effects (min)	21 (10−35)	18 (3−30)	26 (10−60)	13 (5−30)
Time to train women to self-inject (min)	NA	NA	57 (35−90)	39 (10−53)
Medical screening during first visit				
Probability to be tested for pregnancy when seeking hormonal contraceptives	20%	20%	20%	19%
Percentage of women accepting condoms as backup method (health worker-reported)	18%	89%	28%	80%
Number of condoms given as backup method	5	5	5	5
Women’s travel and opportunity costs				
Percentage of women working (formally or informally)	36%	66%	66%	41%
Weighted one-way travel time to the facility (min)	35	27	55	54
Weighted waiting time at the facility/to see a Village Health Team (min)	39	22	50	53
Percentage of clients seeking treatment of side effects				
Average percentage of clients seeking treatment of side effects with DMPA-SC	40%	26%	7%	3%
Average percentage of clients seeking treatment of side effects with DMPA-IM	25%	33%	9%	8%

We estimated health workers’ time costs by multiplying the self-reported time spent with a client for each family planning service by the average salary per minute of the health workers. We included facility-based health worker time costs, valued at the average government salary pay by grade. We valued volunteers’ time using the salary of a nursing assistant. For the Uganda Village Health Teams study, in addition to health workers’ time, we also included time and travel costs for Village Health Teams to reach the communities where they met with women and the travel costs to reach their facility for replenishment of supplies or data reporting.

To estimate costs of medical supplies and tests, we asked health workers to list all medical supplies and tests used in the delivery of each contraceptive method and the percentage of women on whom these were used. We calculated the costs by multiplying the quantity of supplies and pregnancy tests used per client with the corresponding unit price [23], [24] and percentage of women with whom the supplies or tests were used (Table 2, Table 3, Table 4). We also included the costs of the condoms provided as a backup method for women initiating injectable contraception, weighted by the percentage of women accepting condoms.

Costs of treatment of side effects included the costs of health worker time spent on counseling and/or treating a client experiencing side effects, and the drugs they typically prescribed for treating these side effects. We used similar approaches as described above to calculate the costs for health workers’ time. To calculate drug costs, we multiplied each unit price by the total quantity prescribed for each side effect and by the probability of experiencing the side effect over the first 12 months of use based on data from the continuation studies (Table 3). We calculated drug costs for the two most common side effects per injectable type, as reported by health workers. Typical drugs used were ibuprofen, paracetamol and oral contraceptives.

To estimate the costs of medical equipment related to safe disposal of injectable contraceptives, we divided the cost of a safety box by the number of DMPA-SC and DMPA-IM units that could fit in one safety box [25].

We estimated costs of training women to self-inject DMPA-SC under a research study design. These costs included the time spent by study health workers to train clients to self-inject and the costs of training material calculated by multiplying the quantity of each supply by the respective unit cost. We estimated costs for retraining women by multiplying the training costs by the percentage of women who were retrained.

We adjusted costs of training supplies to better reflect current programmatic implementation in Uganda. Namely, we replaced the self-injection training booklet given to women under the research study with a lower-cost, one-page (front-and-back) training instruction sheet with the same information.

We derived travel and time costs for women to receive injectable contraceptives using client-level data collected during method continuation studies [6], [7], [8]. We summed women’s reported travel time to reach the facility, the waiting time to be seen by a health worker (facility-based health worker or a Village Health Team in their community) and the time spent with the health worker to determine the total time spent to receive family planning services. To estimate women’s time costs [26], [27], we multiplied the percentage of women working (formally or informally) by the annual country gross domestic product per capita [28] and the total time spent to receive family planning services.

We calculated roundtrip transport costs per visit to the facility or Village Health Team using the self-reported data. We calculated annual costs for DMPA-SC or DMPA-IM users by multiplying the costs per visit by four when women went to a facility/Village Health Team for all injections. Instead, for self-injecting women, we included the costs of the first visit and the (minor) costs for subsequent visits in case women sought help at the health facility for side effects.

### 2.4 Data analysis

We performed data analysis in Microsoft Excel (Redmond, WA, USA). We converted costs in local currency to 2016 US$ using annual average exchange rates by country; US$1 corresponded to 3419 Ugandan shillings and 595 West African Francs (Burkina Faso and Senegal) [29]. The cost estimates reflect the annual average direct medical and nonmedical costs per client (for four injections). One-way sensitivity graphs show how the estimated average costs change when varying one cost component at a time. We determined the cost variation applying the minimum and maximum values of each cost component based on the primary data we collected. The graphs identify drivers of the variability in the estimated average costs.

## 3 Results

Table 5 presents estimated direct medical and nonmedical costs for DMPA delivery in various settings. Commodity costs accounted for the largest share of the direct medical costs, except under the self-injection research intervention before adjustment, for which client training aid represented the largest costs. Variation in self-reported duration of client visit times, health workers’ salaries, and reported side effects and treatment practices drove differences in the estimated direct medical costs across countries.

**Table 5:** Direct medical and direct nonmedical costs of DMPA-SC injectable contraceptive delivery over four injections (per CYP) by delivery strategy and country (in 2016 US$)^a^

	Burkina Faso		Uganda				Senegal	
	DMPA-SC (facility-based delivery)	DMPA-IM (facility-based delivery)	DMPA-SC (community-based distribution)	DMPA-IM (community-based distribution)	Self-injection (DMPA-SC)	DMPA-IM (facility-based delivery)	Self-injection (DMPA-SC)	DMPA-IM (facility-based delivery)
Contraceptive commodity costs (with syringe)	$3.40	$3.32	$3.40	$3.32	$3.40	$3.32	$3.40	$3.32
Health workers’ time costs (including Village Health Team travel)	$2.99	$2.85	$1.04	$1.08	$0.71	$0.99	$1.73	$1.99
Costs for medical supplies and tests	$0.67	$0.67	$0.23	$0.23	$0.34	$1.03	$0.57	$1.06
Costs for supplies for self-injection training	NA	NA	NA	NA	$3.60/$1.70a	NA	$3.58/$1.68a	NA
Costs of drugs for treatment of side effects	$0.86	$0.54	$0.27	$0.32	$0.07	$0.09	$0.02	$0.05
Waste disposal costs	$0.01	$0.02	$0.01	$0.02	$0.01	$0.02	$0.01	$0.02
Subtotal direct medical costs	$7.92	$7.38	$4.95	$4.97	$8.13/$6.23a	$5.45	$9.31/$7.41a	$6.44
Subtotal direct nonmedical costs (women’s time and travel)	$4.22		$2.74		$1.60	$4.66	$0.97	$3.02
Total direct medical and nonmedical costs	$12.14	$11.60	$7.69	$7.71	$9.73/$7.83a	$10.12	$10.28/$8.38a	$9.46

Abbreviation: NA, not applicable.

a Costs adjusted to reflect replacement of client instruction booklet with client one-page instruction sheet, as implemented in Uganda after the self-injection research study was completed.

We estimated supplies for self-injection training at $3.60 for Uganda and $3.58 for Senegal before adjustment, with the training booklet ($2.00) accounting for the largest share of the costs. The adjusted training aid costs reflect the replacement of the booklet with the one-page instruction sheet ($0.07), and the estimated self-injection training costs for Uganda and Senegal were $1.70 and $1.68, respectively (Table 5).

Total direct medical costs were lowest for community-based distribution by Village Health Teams in Uganda ($4.95 for DMPA-SC; $4.97 for DMPA-IM) because of lower Village Health Teams time costs. Community-based distribution also saved costs for tests and supplies typically used during the first facility visit. In Uganda, the costs were similar for DMPA-IM and DMPA-SC delivered under the same strategy (community-based). We attributed the $0.54 higher cost for four DMPA-SC injections in Burkina Faso relative to DMPA-IM to differences in reported side effects and the associated costs of treatment [14]. The direct medical costs for self-injected DMPA-SC were higher than for facility-based administered DMPA-IM before adjustment ($8.13 for DMPA-SC and $5.45 for DMPA-IM in Uganda; $9.31 for DMPA-SC and $6.44 for DMPA-IM in Senegal). Once we adjusted the costs of client training aids, the difference in the direct medical costs of self-injection compared to DMPA-IM dropped to less than $1.00 (Table 5).

Women’s time and travel costs estimates ranged from $0.97 to $4.66 across countries and delivery strategies (Table 5). The lowest costs were for women who self-injected because most of these women had to visit the health facility only once, for the first injection, and only a small percentage of women returned to the facility for treatment of side effects. The highest costs were for women who received DMPA-IM from a facility in Uganda. Community-based contraceptive delivery in Uganda resulted in lower costs for women than traveling to a facility to receive services.

When we included both direct medical costs and direct nonmedical costs, the adjusted total direct costs of self-injection in Uganda and Senegal were lower than the total direct costs of DMPA-IM delivered by facility-based health workers (Table 5).

**Fig. 1:**
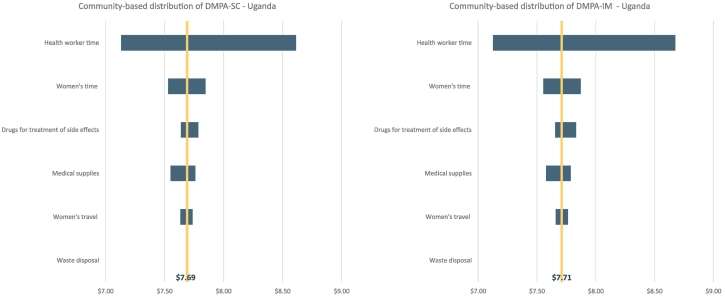
One-way sensitivity analysis for the costs of community-based distribution of DMPA-SC and DMPA-IM in Uganda.

**Fig. 2:**
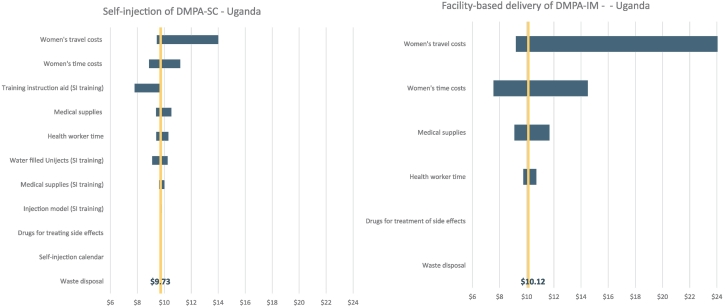
One-way sensitivity analysis for the costs of self-injection of DMPA-SC and provider-administered DMPA-IM in Uganda.

Figs. 1 and 2 and Appendix Fig. A1, Fig. A2 show results from one-way sensitivity analyses and how the changes in each of the cost components affect the cost estimates. Generally, the ranges between the two products are quite similar when they are delivered by the same types of health workers. In Burkina Faso and Uganda, when both DMPA-SC and DMPA-IM are administered by health workers, the cost of provider time is the largest single cost component with the most variability. For the costs of self-injection in Uganda (Fig. 2) and especially Senegal (Fig. A2), women’s travel costs led to the largest variability in costs.

## 4 Discussion

Research studies on DMPA-SC delivery alongside DMPA-IM provided a unique opportunity to analyze the costs of this new contraceptive technology across different delivery strategies. In both Uganda and Burkina Faso, the costs for DMPA-SC were similar to the costs for DMPA-IM when delivered under the same strategy; hence, there is no clear cost advantage of one product over the other. In Uganda, community-based distribution of DMPA injectable contraception resulted in lower costs for both service delivery (direct medical costs) and for women (direct nonmedical costs) when compared to facility-based DMPA-IM delivery. Bringing contraceptive services closer to women by leveraging and allowing community health workers to provide short-term contraceptives may help increase access to and uptake of contraceptives — especially in rural areas, where women may face higher financial barriers that limit their ability to travel to a facility to receive services [30]. Our sensitivity analysis showed that when health workers administer DMPA-SC and DMPA-IM, the cost of health worker time is an important driver of total costs. This underscores the potential for family planning interventions that enable task shifting to help reduce health system costs.

The option to self-inject may further reduce financial and logistical barriers for women. Women’s travel and time costs accounted for 32% to 46% of total costs when health workers administered the injections compared to 9% to 20% of total costs when self-administering. As the one-way sensitivity analysis for these Senegal and Uganda self-injection studies showed, there was a much wider range in women’s travel costs over the four injections for women who were receiving DMPA-IM injections from providers than for women who were self-injecting; the relative cost savings for women who self-inject will also vary widely depending on the distance between their home and the facility and, hence, their travel costs.

The upfront costs to train women to self-inject can be high, as observed under the research study. As the sensitivity analysis showed, the training aid was an important driver of costs. Simple modifications to the training aid used can reduce client training costs, likely without affecting the quality and effectiveness of the training. As self-injection is offered and scaled up in more settings, these cost drivers can be reduced to make self-injection training more affordable and sustainable.

Our cost estimates are comparable with previous study results, which found facility-based delivery of DMPA-IM to cost between $8.50 and $20 per CYP [8], [9]. A study conducted in Ghana estimated the average contraceptive delivery costs at $25.40 per CYP [10]. This study included direct medical and direct nonmedical costs related to administrative staff time, office equipment, staff training and programmatic needs. Another study, in Ethiopia, found that the average programmatic cost to provide injectable contraceptives via a community-based social marketing program was $17.91 per CYP, of which $2.96 went to direct service provision [12]. Evaluation of direct costs for Kenya revealed that DMPA-SC delivered in facilities costs slightly more than DMPA-IM: $8.19 per CYP and $7.07 per CYP, respectively [13].

This study had several limitations. First, the study sites that we used for the costing analysis were not representative of their districts or of the country in which the study was set, and the sample sizes were small. The need to select facilities from those involved in the pilot introduction of DMPA-SC or the research study of self-injection drove this limitation. Second, self-reported estimates of health worker and client resources may be inaccurate due to recall bias. In addition, this analysis looked at the annual costs of DMPA (four injections); however, women’s 12-month use of injectable contraception across methods and delivery modalities may differ. Finally, we did not include some delivery or programmatic costs in this study, such as costs for training and supervising health workers to deliver injectables, facility operational and management costs, and supply chain costs.

The following are the supplementary data related to this article.

Fig. A1 One-way sensitivity analysis for the costs for facility-based delivery of DMPA-SC and DMPA-IM in Burkina Faso. Fig. A1

Fig. A2 One-way sensitivity analysis for the costs for self-injection of DMPA-SC and facility-based delivery of DMPA-IM in Senegal. Fig. A2
