# Supplementary material for: Costs of administering injectable contraceptives through health workers and self-injection: evidence from Burkina Faso, Uganda, and Senegal
Source: Contraception. 2018 Nov;98(5):389–95. doi: 10.1016/j.contraception.2018.05.018 (PMC6197836; doi:10.1016/j.contraception.2018.05.018)

Figure A1. One-way sensitivity analysis for the costs for facility-based delivery of DMPA-SC and DMPA-IM in Burkina Faso

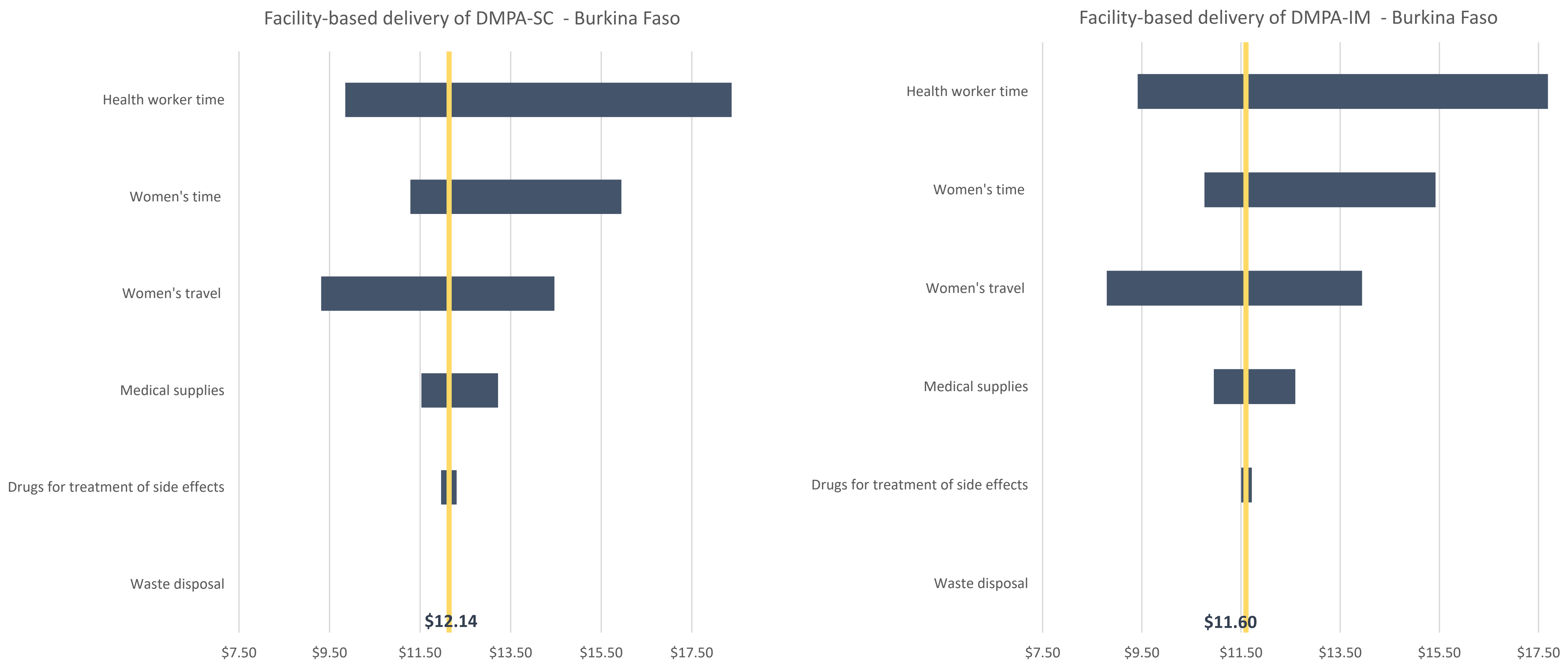

Supplement: Fig. A1 — One-way sensitivity analysis for the costs for facility-based delivery of DMPA-SC and DMPA-IM in Burkina Faso. [file mmc1.pdf]
